# Ethical Development of Artificial Amniotic Sac and Placenta Technology: A Roadmap

**DOI:** 10.3389/fped.2021.793308

**Published:** 2021-12-08

**Authors:** E. J. Verweij, Lien De Proost, Judith O. E. H. van Laar, Lily Frank, Sylvia A. Obermann-Borstn, Marijn J. Vermeulen, Sophie van Baalen, M. Beatrijs van der Hout-van der Jagt, Elselijn Kingma

**Affiliations:** ^1^Division of Fetal Therapy, Department of Obstetrics, Leiden University Medical Center, Leiden, Netherlands; ^2^Department of Obstetrics and Gynaecology, Erasmus Medical Center (MC), Rotterdam, Netherlands; ^3^Department of Neonatology, Erasmus Medical Center (MC), Rotterdam, Netherlands; ^4^Department of Medical Ethics, Philosophy and History of Medicine, Erasmus Medical Center (MC), Rotterdam, Netherlands; ^5^Department of Obstetrics and Gynaecology, Máxima Medical Centre, Veldhoven, Netherlands; ^6^Faculty of Electrical Engineering, Eindhoven University of Technology, Eindhoven, Netherlands; ^7^Department of Industrial Engineering and Innovation Sciences, Eindhoven University of Technology, Eindhoven, Netherlands; ^8^Care4Neo, Rotterdam, Netherlands; ^9^Rathenau Instituut, The Hague, Netherlands; ^10^Department of Biomedical Engineering, Eindhoven University of Technology, Eindhoven, Netherlands; ^11^Department of Philosophy, King's College London, London, United Kingdom

**Keywords:** artificial placenta, artificial womb, ectogenesis, ectogestation, ethics, research ethics, value sensitive design, perinatal life support

## Abstract

In this paper we present an initial roadmap for the ethical development and eventual implementation of artificial amniotic sac and placenta technology in clinical practice. We consider four elements of attention: (1) framing and societal dialogue; (2) value sensitive design, (3) research ethics and (4) ethical and legal research resulting in the development of an adequate moral and legal framework. Attention to all elements is a necessary requirement for ethically responsible development of this technology. The first element concerns the importance of framing and societal dialogue. This should involve all relevant stakeholders as well as the general public. We also identify the need to consider carefully the use of terminology and how this influences the understanding of the technology. Second, we elaborate on value sensitive design: the technology should be designed based upon the principles and values that emerge in the first step: societal dialogue. Third, research ethics deserves attention: for proceeding with first-in-human research with the technology, the process of recruiting and counseling eventual study participants and assuring their informed consent deserves careful attention. Fourth, ethical and legal research should concern the status of the subject in the AAPT. An eventual robust moral and legal framework for developing and implementing the technology in a research setting should combine all previous elements. With this roadmap, we emphasize the importance of stakeholder engagement throughout the process of developing and implementing the technology; this will contribute to ethically and responsibly innovating health care.

## Introduction

Worldwide, extreme prematurity at the limit of fetal viability is a main cause for perinatal mortality and morbidity (1, 2). In high-income countries most of the extremely premature infants receive highly specialized neonatal care at birth. This care approach includes respiratory support, intubation, medication, and eventual resuscitation at birth, which is invasive and often painful for the infant. Overall, the mortality rate is high but varying even between high income countries (3). Some infants survive with severe long term disabilities and some experience, sometimes life long,side effects of the neonatal intensive care treatment (3). In the past decades, much research has aimed at improving artificial support at birth for these infants, but without substantial result for long-term outcomes (4, 5). One recent technology that has attracted much attention is the artificial amniotic sac and placenta technology (AAPT). AAPT is a technology that may in the future facilitate a radically different alternative treatment option to conventional neonatal care. Other common terminology for AAPT in the literature includes artificial womb technology (AWT), EXTrauterine Environment for Neonatal Development therapy (EXTEND) (6, 7), *ex-vivo* uterine environment therapy (EVE) (8), biobag, and perinatal life support (PLS) (9)—all referring to similar treatment options and corresponding technologies.

AAPT mimics the function of the amniotic sac, the amniotic fluid and the placenta (6, 8). It thereby aims to keep the fetus/baby in what is effectively a fetal physiological state. This means (or so it is hoped) that organs, most notably the lungs, can continue their development/maturation, avoiding main causes of very early neonatal death or severe morbidity in infants born at the limits of viability. If successful, this approach would offer several advantages over the use of a standard neonatal incubator But it is not a viable alternative for an entire pregnancy. *In vivo* pregnancy will remain indispensable for embryo implantation and early development of the embryo and fetus as the technology requires a developed fetal heart and cardiovascular system with substantial capacity, making its use only viable after 20 weeks. Nor should we forget that pregnancy, and maternal-fetal interaction, consists of more than providing a developing fetus with oxygen and nutrients. The ultimate goal of AAPT is not to provide an alternative to normal pregnancy, but to increase the survival rate of infants born at the edge of viability; to limit complications and severe disabilities; and to improve the quality of life of extremely premature infants (10). The technology shows promising results in animals, but has not yet been tested on humans (6–8). It is important to also bear in mind that the human pregnancy will differ from the animal system as that is a healthy pregnancy terminated for the study whereas in clinical practice it is a spontaneous preterm birth with underlying pathology.

But the development and implementation of AAPT comes with ethical challenges that need to be addressed, taking account of the viewpoints of different stakeholders: society, patients, families and caregivers for a range of different societies In this paper, we consider four elements of attention in the ethical development and implementation of AAPT: (1) framing and societal dialogue; (2) value sensitive technological design, (3) research ethics and (4) ethical and legal research, all of which should be incorporated into an adequate moral and legal framework. Stakeholder engagement is of major importance to continue developing and implementing the innovative technology of the artificial placenta in an ethically and socially responsible way.

## Ethical Development of AAPT Toward the First In-Human Trial

### Framing and Societal Dialogue

#### Stakeholder Involvement

Development and implementation of AAPT can only succeed if the technology is socially accepted. This is contentious terrain; current media-discussions tend to be heated and may induce concern (11). AAPT cannot be designed and developed without extensive dialogue and informed debate in which all relevant stakeholders are involved. Stakeholders include patients, families and caregivers, ideally in a range of different societies. But because of widespread anxiety surrounding, and cultural resonance of reproductive technologies in general—and “artificial wombs” in particular—all of society should be considered a relevant stakeholder. Hence, members of the general public should also be included in the dialogue. Stakeholders should be consulted on their perspectives on the technology and its use in clinical practice *throughout* technological development and human research, and not just at the point of implementation. This requires a societal dialogue that includes a wide and diverse audience, in the form of a series of moderated dialogues with different groups of stakeholders and members of the public. The aim of such a dialogue would be to inform about the opportunities and uncertainties of the technology, and possible societal and ethical issues that surround it. In the dialogue, participants from a different range of societies would be invited to discuss their hopes, questions, wishes and concerns on the clinical application. This approach aims to stimulate a collective process of opinion forming and reflection. This method does not result in a description of “the societal consensus” on this issue, as participants do not a constitute a representative sample of society and the aim of the dialogues is not to reach consensus on the topic. However, it does provide deep and detailed insights in the different perspectives of stakeholders and members of society, the arguments they use to substantiate their view, under which conditions they think development and use of the technology is acceptable and which values are important to protect. The values that emerge from the societal dialogue should play a role in all other elements of ethical development that we discuss (12).

#### Framing and Terminology

Crucial for a dialogue of high quality is a correct understanding of AAPT. In relation to that, a correct, balanced and well-framed terminology is important.

AAPT has often been described as artificial womb (technology), which invokes strong social anxieties. This term is misleading. The technology does not mimic the entire complex function of the womb, and—perhaps because of its literary origins—it invokes highly unrealistic ideas of what the technology is capable of. This leads to concerns (or the belief, etc.) that AAPT is being considered as a replacement for an entire pregnancy (or even for women!)—as opposed to (in reality) a replacement for neonatal incubation at the edge of viability. Importantly, AAPT will also not be capable to take over motherhood and bonding. Whilst societal discussion may need to air and engage these wider cultural anxieties, it should also encourage a debate based on a realistic image of the possibilities and limitations of AAPT generates. This can be facilitated by using terminology and imagery that encourages a correct understanding of the technology.

Another important factor for societal dialogue is that different perspective are given the opportunity and space to be heard and considered, and that all participants are equal partners in the dialogue. Different participants can have different views of what is at stake and what should be the main issue of the dialogue. Scientific experts should not just be informing the public, but also be learning from other stakeholders about, for example, their experience and perspective (13).

### Value Sensitive Design

Value sensitive design is gaining more and more ground. Friedman et al. provide the following definition of the concept: “Value Sensitive Design is a theoretically grounded approach to the design of technology that accounts for human values in a principled and comprehensive manner throughout the design process” (14). For developing and implementing technological innovations like AAPT, value sensitive design is essential. Since the technology may not only drastically change high-risk newborn care, but would also influence a range of normatively valanced social relations and perceptions, such as our perception of pregnancy, childbirth and women, as well as our ideas about bonding, motherhood, and neonatal care, it is especially important to take into account societal values *throughout* the design process.

For example, throughout the technological design process, choices will have to be made—such as choices about the accessibility, visibility and levels and kinds of interaction facilitated between the fetus/baby and the caregivers/parents. Such choices embody, but also facilitate, certain kinds of framings and normatively valanced relations. To give but a simple example: should the AAPT look plastic, transparent and “high tech,” or red, warm, homely and womb-like? Such choices may be constrained by technological ability or fetal physiology—but there is also room for responding to social and normative direction. This requires ongoing and iterative stakeholder engagement.

As is the case with many other innovations, this technology has soft as well as hard impacts on a societal level (15, 16). On the one hand, hard impacts of implementing AAPT in clinical practice could be the increase in survival and survival without disabilities for extremely premature infants, the increase in health care costs (but also eventual decrease because there will be less lives with severe long-term disabilities), and changes in the NICU infrastructure. Possibly, another hard impact concerns the psychosocial development of the child. Although the technology obviously strives to provide optimal support of normal development, its actual impact is not yet known. On the other hand, there are also soft impacts of the implementation of this technology. Swierstra explains these kinds of impacts as follows: “In brief: (soft) impacts are qualitative rather than quantitative; the core values at stake are unclear or contested rather than clear instances of harm; and the results are co-produced by the user rather than being caused solely by the technology” (16). Soft impacts of AAPT could include the changing relationship with and attitudes toward the fetus and childbirth, a changing concept of viability, new ideas on parent-infant connectedness and parental responsibility. Furthermore, the above-mentioned impacts could result in increased moral pressure to “opt in” on this technology. These hard and soft impacts should be discussed as part of the societal dialogue and the perspectives from the society and stakeholders should inform in the design of the technology.

A significant barrier in regards to the value sensitive design of AAPT is uncertainty. Both the long- and short-term consequences for the child, mother and other family members are unknown. Survival after AAPT will be known relatively early in the study process, but for example gaining knowledge about neurodevelopmental outcomes will take years. Besides that, AAPT implies that there will be limitations in physical contact between the parent(s) and the infant after the caesarian. This limited parental-infant contact will be for several weeks, during the time that the infant will receive AAPT-based treatment. It is not known yet what effects this separation may have on the parent(s) as well as the infant. However, we know from research that separation can be of important influence for infant development (17). There could be cognitive, social and or emotional effects on the (development of the) infant, or it could affect the parental-infant attachment. Value sensitive design requires ongoing interaction and feedback loops with stakeholders as part of the social dialogue—taking into account all these uncertainties.

### Human Research Ethics

Human research ethics for AAPT is challenging and in need of thorough study. We will highlight a few important elements: patient selection, informed consent and therapeutic misconception.

In general, several challenging questions are important for first-in-human studies of AAPT: (a) *when* exactly the technology can be regarded as safe enough to start first-in-human research, (b) *who* are the eventual study participants on which the technology can be tested, and (c) *how* to assure ethical decision-making possibilities for the study participants. The results from animal studies do not imply similar results for future human studies. Human studies with long-term follow-up are needed before implementing AAPT in clinical practice. Moreover, clinical research must be conducted according to the ethical principles of proportionality and subsidiarity; in the context of first-in-human studies with AAPT this means that the attributable risks to the group of pregnant women and extremely premature infants should be minimized and that the risks and burdens should be commensurate with the potential benefits of the technology.

Given the risk for adverse outcomes for the infants involved, there should be a low threshold for termination of any study protocol if outcomes deviate from those generated from the current standard of care (incubation). The studies should not be stopped early until adequate long-term follow-up is performed. Once the therapy is accepted as standard of care, it will be harder to stop it if problems with neurodevelopmental outcome is detected. Similarly, while it is true that information such as differences in neurological outcome will take many years to generate, care should be given to identify appropriate short-term outcomes to allow for a more rapid progression of research if clinically indicated given the substantial limitations of our current interventions for extremely premature infants.

#### Selection of the First Study Participant

Carefully selecting study participants for first-in-human research with AAPT is of major importance. Not every pregnant person who is in a situation of (threatening) extremely premature birth must automatically qualify as a study participant; certain requirements for study participants can be formulated so that the recruitment and inclusion process is ethically responsible. To this end, we propose the following inclusion criteria for a woman (and her partner): (a) they have enough time to decide whether or not to participate in the study, (b) their decision is supported by high-quality counseling and they are able to understand and weigh the pros and cons of participating in the study; they are as much as possible aware of the uncertainties involved; (c) they are in a situation in which a cesarean is *per se* clinically indicated; and (d) they are in a situation in which for the mothers health it is much better to be no longer be pregnant (for example maternal complications like pre-eclampsia). Another important question is whether the first participants should be selected from a population that would be considered “pre-viable” under existing technologies.

To transport the fetus from the womb to the AAPT is a very specialized and timely operation. The transition toward respiration should be prevented. The subject should be transferred without being able to breath. During vaginal labor the transition already starts and the moment of transfer is much more difficult to plan what is inducing risks for a safe transfer. For this reason, it is clear that (at first) AAPT is only possible when the fetus has left the maternal womb through a cesarean. Every cesarean comes with accompanying maternal health risks, physical as well as mental (18, 19). Moreover, a cesarean comes with increasing risks in eventual subsequent pregnancies (18). For this reason it would not be proportional to do a cesarean specifically for transfer to the AAPT in the first clinical trials.

#### Informed Consent

Ensuring that the autonomy of the research participants is respected is a foundational requirement of ethical research. Barring a few exceptions (e.g., research on those who are incapable of giving consent, but may still derive a benefit from the research), informed consent is one of the ways in which this respect is instrumentalized. The requirement is encoded in research ethics regulations (20).

Developing an adequate informed consent procedure for the first-in-human studies of AAPT is challenging. For example, one complication is that the pregnant woman is the consenting research participant, acting in multiple capacities. She is a research participant in her own right, as it is her body that will be operated on. But the subject of AAPT is also exposed to interventions and risk of harm, and could be considered a research subject, in its own right. Then, as is the case with research involving fetuses, neonates, and children, it is the parent or prospective parent that has to decide on behalf of the fetus, as a surrogate decision maker. The role of the pregnant woman's partner/co-parent (if she has one) in the informed consent process also needs to be clarified. In clinical research on pregnant women the widely accepted ethical standard is that there is no requirement or need for the partner to play a role in the consent process if the woman does not want them to (21). Is the case of AAPT different in any ethically significant way?

#### Therapeutic Misconception

One particular point of attention in the development of the first-in-human studies of AAPT is the therapeutic misconception. This is defined as research participants' persisting assumption that decisions relating to research interventions are made on the basis of their individual therapeutic needs, despite clear information to the contrary. However it is also more widely used for misunderstandings that participants of clinical trials might have (21, 22). Medical practice and research are often intertwined and it may sometimes be difficult to draw the line between both; this will be particularly applicable to the first in-human AAPT. Patients who could be participating are aware of the worrying prognosis on the conventional NICU and may choose for AAPT with inflated hopes that the results will be better and/or fail to realize that the goal of the research is primarily the generation of knowledge for the benefit of future patients (23). The study design for the first-in-human studies of AAPT should take measures to reduce therapeutic misconception, such as careful counseling by “independent” doctors.

### Ethical and Legal Research

An important part of the moral and legal framework is a robust ethical and legal analysis. The concepts “embryo,” “fetus,” and “neonate” may no longer suffice as moral, social and legal categories involved in the development of an infant once AAPT is being developed. Romanis proposes calling the subject of AAPT a “gestateling” (24). Kingma and Finn explain: “Fetuses and gestatelings (however much supported) are not yet “born-by-physiology-change,” and have fetal physiology and characteristics; neonates, by contrast, are “born-by-physiology-change,” and have neonatal physiology and characteristics—again, however much supported. Gestatelings share with neonates, in contrast to fetuses, that they are “born-by-location-change”—and hence reside outside rather than inside the maternal body” (11). This is likely to make a difference for ethics and law, but the ethical-legal consequences have not yet been worked out and may differ between jurisdictions. Embryos, fetuses and neonates differ in their legal status, and their moral status is up for debate. The moral and legal status of the subject in the AAPT may have important consequences for questions such as the formal rights and role of the co-parent; legal research possibilities; and the role of viability in abortion law.

## Moral and Legal Framework

Eventually, a moral and legal framework for AAPT must be developed taking into account all previous elements. The discussed elements—societal dialogue with stakeholders, value sensitive design, research ethics, and the moral and legal status of the subject in the AAPT—should be integrated in a robust ethical and legal framework to guide socially acceptable ethical development of AAPT.

## Conclusion

In this article, we put forward four important, intertwined elements for the ethical development of AAPT. Our attempt was not to discuss all ethical challenges in detail however, it was to provide a possible way forward for responsibly innovating care. In conclusion, we want to underline the importance of a timely dialogue and stakeholder engagement and studying the soft impacts of the innovation. The road toward implementation of the AAPT will bring about many uncertainties. Together with stakeholders, it should be reflected on how to cope with these uncertainties in the most delicate way such that the results can feedback into technological and research design. A critical view, stakeholder engagement and robust ethical analysis are needed in every step of the roadmap to balance the pros and cons of AAPT. If the first results of AAPT are positive, a next roadmap should be made in time for the implementation of AAPT in clinical practice.

## Data Availability Statement

The original contributions presented in the study are included in the article/supplementary material, further inquiries can be directed to the corresponding author/s.

## Author Contributions

EV, EK, and LD wrote the main manuscript text based on a discussion session with all other authors. All authors contributed to the article and approved the submitted version.

## Funding

EK time is funded by the European Research Council (ERC) under the European Union's Horizon 2020 research and innovation programme (grant agreement no. 679586).

## Conflict of Interest

MBvdH-vdJ is shareholder in Juno Perinatal Healthcare BV, the Netherlands. The remaining authors declare that the research was conducted in the absence of any commercial or financial relationships that could be construed as a potential conflict of interest.

## Publisher's Note

All claims expressed in this article are solely those of the authors and do not necessarily represent those of their affiliated organizations, or those of the publisher, the editors and the reviewers. Any product that may be evaluated in this article, or claim that may be made by its manufacturer, is not guaranteed or endorsed by the publisher.
